# Maternal obesity, diet, and the gut microbiome: implications for offspring cardiometabolic programming

**DOI:** 10.3389/fnut.2026.1909112

**Published:** 2026-09-24

**Authors:** Alireza Jahan-Mihan, Shekoufeh Salamat, Reniel Nodarse, Rachael Shangraw, Corinne Labyak, Jill Snyder, Kristin Berg, Jamisha Leftwich

**Affiliations:** 1 Department of Nutrition and Dietetics, University of North Florida, Jacksonville, FL, United States; 2 Department of Nutritional Sciences, Temerty Faculty of Medicine, University of Toronto, Toronto, ON, Canada

**Keywords:** cardiometabolic programming, dietary patterns, gut microbiome, maternal obesity, offspring health, pregnancy, probiotics

## Abstract

Maternal obesity is associated with adverse pregnancy and long-term offspring outcomes. The maternal gut microbiome may contribute through altered microbial functions, barrier integrity, immune signaling, and metabolite production. Pregnancy itself entails microbial and metabolic remodeling, but obesity and obesogenic dietary patterns may modify these adaptations. Evidence from human cohorts and animal models links maternal obesity with context-dependent differences in microbial composition and function, reduced butyrate-producing capacity, altered bile acid and short-chain fatty acid profiles, and heightened inflammation. These changes may influence placental function and early-life microbial assembly through delivery-related exposures, breastfeeding, human milk oligosaccharides, and shared environmental and dietary factors. Maternal diets high in saturated fat and refined carbohydrate tend to be associated with less favorable microbial and cardiometabolic profiles, whereas fiber-rich, plant-diverse dietary patterns may support short-chain fatty acid production and metabolic resilience. However, causal inference is limited by heterogeneity, confounding, and inconsistent findings. Probiotic, prebiotic, and synbiotic interventions show potential, but clinical results—particularly for gestational diabetes prevention—remain mixed. This narrative review integrates evidence on diet–microbiome interactions in maternal obesity, microbial metabolites and immune pathways, early-life colonization, and implications for offspring obesity and cardiometabolic risk. Overall, the maternal microbiome is a plausible but incompletely validated target; well-powered longitudinal studies and pregnancy-specific randomized trials are needed before precision microbiome strategies can be incorporated into routine care.

## 1 Introduction

Obesity has become one of the most pressing public health challenges of the 21st century, with global prevalence continuing to rise among women of reproductive age. Maternal obesity, commonly defined using a pre-pregnancy body mass index (BMI) of 30 kg/m^2^ or greater, is associated with increased risks of gestational diabetes mellitus (GDM), hypertensive disorders, cesarean delivery, and excessive gestational weight gain (GWG) (1–3). Beyond maternal complications, offspring born to mothers with obesity have higher risks of altered growth trajectories, obesity, insulin resistance, and cardiometabolic disorders later in life. These observations support the concept of developmental programming, wherein maternal metabolic and environmental factors shape fetal and infant health with long-lasting consequences (4).

In recent years, the gut microbiome has emerged as a plausible biological link between maternal obesity and maternal and offspring outcomes. The maternal gut microbiota may contribute to energy metabolism, immune regulation, intestinal barrier integrity, and microbial metabolite production during pregnancy (5). In this review, “microbiota” refers to microbial communities present in maternal, milk-associated, or infant sites, whereas “microbiome” is used more broadly to include microbial composition, genes, functions, and metabolic outputs. Maternal obesity has been associated with context-dependent changes in microbial composition and function, systemic inflammation, altered short-chain fatty acid production, and increased endotoxin-related signaling; however, much of the human evidence is observational and does not establish causality (6).

Maternal diet is a central and modifiable determinant of both cardiometabolic health and microbial function during pregnancy. Diets high in saturated fat and refined sugars may reinforce dysbiotic and pro-inflammatory patterns, whereas fiber- and polyphenol-rich dietary patterns can support saccharolytic fermentation, short-chain fatty acid production, and metabolic resilience (7, 8). Probiotic, prebiotic, and synbiotic interventions are also being investigated, but their effects appear to depend on strain, dose, background diet, timing, and maternal metabolic status (9).

Important gaps remain in understanding how maternal adiposity and diet relate to microbial function during pregnancy, whether microbial metabolites or immune signals contribute to placental and offspring outcomes, and which interventions are both effective and safe. Previous reviews have often considered maternal obesity, gestational diabetes, pregnancy-associated microbiome variation, or infant colonization separately. This narrative review brings these areas together by examining maternal diet and obesity in relation to microbial functions and metabolites, placental and lactational pathways, early-life microbial assembly, and offspring cardiometabolic programming. Particular attention is given to the strength and source of the evidence, the distinction between association and causation, and the boundary between established dietary and lifestyle care and microbiome-directed strategies that remain investigational.

## 2 Methodology

This narrative review synthesizes evidence on maternal obesity, diet, the gut microbiome, and maternal and offspring health. A structured literature search was used to identify relevant evidence while retaining the interpretive scope appropriate to a narrative review.

PubMed, Scopus, Web of Science, and UNF OneSearch were searched for English-language publications from January 2000 through August 2025. Searches combined terms relating to maternal or gestational obesity, pre-pregnancy body mass index, gestational weight gain, pregnancy, gut microbiota or microbiome, microbial metabolites, diet, probiotics, prebiotics, synbiotics, placenta, lactation, vertical transmission, infant colonization, and offspring health. Boolean operators and database-specific subject headings were used as appropriate, and the reference lists of relevant articles and reviews were examined for additional studies.

Eligible publications included human observational studies, pregnancy-specific clinical trials, animal studies, and mechanistic studies relevant to maternal metabolic status, microbial composition or function, placental or lactational pathways, infant microbial development, or offspring outcomes. Reviews were used to identify and contextualize primary literature. Conference abstracts, editorials, and reports without sufficient scientific detail were not used as principal evidence. Titles and abstracts were reviewed for relevance, followed by examination of the full text of potentially informative publications.

For each included study, attention was paid to study design, population, timing of sampling, maternal metabolic status, dietary exposure or intervention, microbiome method, microbial or metabolite outcome, clinical outcome, and major limitations. The evidence was not subjected to a formal risk-of-bias instrument. Instead, greater interpretive weight was placed on pregnancy-specific randomized trials and prospective human studies with repeated sampling than on cross-sectional associations, animal experiments, or studies in non-pregnant adults. Sample size, confounding, sequencing method, analytical approach, intervention fidelity, and consistency across studies were considered when drawing conclusions.

Because the literature is heterogeneous in design, population, exposure, and outcome, findings were synthesized narratively rather than pooled quantitatively. The synthesis was organized around pregnancy-related microbial variation, maternal obesity and diet, microbial metabolites and host signaling, placental and lactational pathways, early-life microbial assembly, offspring outcomes, and nutritional or microbiome-targeted interventions. Generative artificial intelligence was not used to identify, select, appraise, extract, or interpret studies. Its use was limited to editorial assistance with language and organization after the scientific content had been developed; all revisions were reviewed and approved by the authors.

## 3 Maternal obesity and gut microbiome

The maternal gut microbiome may contribute to maternal metabolism and developmental programming through metabolic, immune, endocrine, placental, and lactational pathways. Its composition and function are associated with maternal metabolic status and may influence fetal and infant exposures (10–13). Maternal obesity has been associated with context-dependent differences in gut microbial composition and function, but findings are heterogeneous and do not establish a universal dysbiotic signature or direct transmission of metabolic traits to offspring (14).

Maternal obesity and obesogenic dietary patterns may interact with pregnancy-related microbial remodeling. Proposed pathways include reduced butyrate-producing capacity, altered short-chain fatty acid and bile-acid metabolism, impaired barrier integrity, and endotoxin-related immune signaling. These changes could influence placental oxidative stress and maternal-fetal nutrient signaling, but direct causal evidence in humans remains limited (7, 15–17).

Maternal diet is a key modulator of microbial composition and function. High-fat and high-sugar dietary patterns have been associated with microbiome changes, although effects vary by dietary fat source, host phenotype, and experimental model. Fiber-rich and plant-diverse diets are more consistently associated with microbial richness and saccharolytic fermentation, providing a plausible route to improved metabolic resilience (18–20).

### 3.1 Natural microbial changes during pregnancy

Pregnancy-related remodeling of the gut microbiome is not uniform across cohorts. Some longitudinal studies report shifts in community structure and metabolic potential across gestation, whereas others find greater stability after accounting for diet, adiposity, medication use, geography, and analytical methods (12, 21, 22). Thus, there is no single microbial signature of healthy pregnancy. Accordingly, the term “dysbiosis” is used cautiously to describe context-specific microbial imbalance or functional disruption, rather than a single universal pregnancy- or obesity-related microbial profile.

Several studies have reported third-trimester increases in *Proteobacteria* and *Actinobacteria* and changes in pathways related to energy metabolism and inflammation (12). These findings have been interpreted as compatible with the physiological insulin resistance and immune adaptation of late pregnancy, but they should not be generalized to all populations.

Likewise, Tang et al. (23) reported changes in alpha and beta diversity are inconsistent. Some cohorts show lower within-sample diversity and greater between-person variability later in pregnancy, whereas others show limited temporal change (21, 22). Differences in sampling frequency, sequencing platforms, diet, antibiotic exposure, and baseline body mass index probably contribute to this heterogeneity.

Functional changes may be more informative than taxonomic shifts alone. Enrichment of microbial pathways involved in carbohydrate utilization, lipid metabolism, and energy harvest has been described during pregnancy, although the direction and clinical significance of these changes remain uncertain (12, 24).

Pregnancy hormones, gastrointestinal motility, diet, and immune tolerance may all shape microbial ecology. These factors are interrelated, making it difficult to attribute observed microbiome differences to gestation itself without well-characterized longitudinal controls.

### 3.2 Impact of maternal obesity on gut microbiota

Maternal obesity may modify pregnancy-related microbial adaptation, but findings vary by population and method. Studies have reported differences in taxa and microbial functions associated with maternal body mass index, insulin resistance, and gestational weight gain; however, no reproducible obesity-specific taxonomic signature has been established (13, 14, 25). In particular, taxa such as *Roseburia* should not be classified uniformly as pro-inflammatory because their associations are context-dependent and often reflect strain- and function-level differences (23).

The *Firmicutes*-to-Bacteroidetes ratio has frequently been discussed in obesity research, but it is not a reliable standalone biomarker of maternal obesity or metabolic risk. Results are inconsistent across studies, and relative-abundance data are compositional, so apparent changes in one group can reflect changes elsewhere in the community (26, 27). Emerging work also links maternal adiposity with one-carbon metabolism and microbial B-vitamin pathways, but current evidence does not establish that obesity routinely causes folate or vitamin B12 deficiency through the microbiome (28).

Maternal obesity is also associated with differences in infant gut microbiota and human milk composition, but these relationships are influenced by delivery mode, intrapartum antibiotics, feeding practices, gestational diabetes, maternal diet, and the shared postnatal environment (14, 27, 29). Accordingly, observed infant microbial profiles should be interpreted as associations rather than evidence that an altered community is directly transmitted or inevitably produces later disease.

#### 3.2.1 Inflammatory pathways and immune modulation in maternal obesity

Maternal obesity has been associated with impaired gut barrier function, low-grade inflammation, and endotoxin-related innate immune signaling, although most pregnancy-specific human evidence remains observational. Metagenomic studies in pregnant women with overweight or obesity have identified associations among diet, microbial function, insulin resistance, lipid measures, and inflammatory status (15). Cohorts that include both obesity and gestational diabetes have also reported microbial patterns associated with metabolic and inflammatory phenotypes, but the independent contributions of adiposity, hyperglycemia, diet, and the microbiome remain difficult to separate (30). These findings support biological plausibility but do not establish microbial changes as the primary driver of placental or offspring outcomes.

At the maternal–fetal interface, inflammation and oxidative stress may connect maternal metabolic status with placental function. Higher inflammatory activity in gestational diabetes has been associated with impaired placental fatty-acid beta-oxidation, while maternal obesity has been associated with altered placental nutrient transport and TLR4-related responses (17, 31, 32). These studies support biologically plausible crosstalk among maternal metabolism, the gut microbiome, and the placenta, but they do not demonstrate that microbiome alterations are the sole or primary cause.

#### 3.2.2 Microbial metabolites and metabolic signaling in maternal obesity

Microbial metabolites have been proposed as one link between diet, the gut microbiome, and maternal cardiometabolic health. Short-chain fatty acids (SCFAs) signal through G protein-coupled receptors and histone deacetylases and can influence gut barrier integrity, inflammatory tone, and glucose metabolism. In pregnant women, circulating and fecal SCFAs have been associated with anthropometric and glycemic measures, although cross-sectional associations cannot determine directionality or mediation (33). Metagenomic findings in women with overweight or obesity further suggest reduced butyrogenic potential and altered carbohydrate metabolism in relation to insulin resistance and lipid profiles (15).

Bile acids provide another potential pathway because microbial biotransformation of bile acids can affect FXR- and TGR5-related signaling, glucose regulation, and lipid metabolism. Studies in gestational diabetes report associations between maternal microbial profiles, bile-acid-related metabolism, and glycemic status, while animal and observational work indicates that obesogenic diets can disrupt pregnancy-related microbial functions and offspring colonization (34, 35). These data support a microbiome–metabolite hypothesis, but pregnancy-specific causal evidence remains limited.

#### 3.2.3 Placental-microbiome crosstalk in maternal obesity

Differences in the maternal gut microbiota and placental stress markers can coexist in obesity. Human placental studies have linked maternal obesity with oxidative stress and redox imbalance that correlate with features of the maternal intestinal microbiota (16). Placental nutrient transporter expression and maternal fatty-acid profiles also vary by maternal obesity and infant size category (31). Together with metagenomic associations between microbial function and maternal insulin resistance (15), these findings are consistent with a possible gut-placenta axis involving inflammatory signaling, metabolite exposure, and altered nutrient handling. They do not establish that microbiome alterations mediate placental dysfunction in humans.

#### 3.2.4 Epigenetic and developmental programming mechanisms

Maternal adiposity and gestational weight gain have been associated with DNA methylation differences in cord blood and placenta, including loci related to infant adiposity and metabolic outcomes (36–38). Newborn methylation-age measures have also been associated with later weight trajectories (39). These findings are consistent with developmental programming, but they do not establish that microbiome changes caused the observed epigenetic marks.

Microbial metabolites and immune signaling may also contribute to neurodevelopmental programming, but much of the mechanistic evidence comes from animal models. In a macaque model of maternal obesity, cell-free fetal DNA methylation patterns were associated with brain and behavioral measures (40), and a mouse model linked high-fat-diet-induced maternal immune activation with altered tryptophan–kynurenine metabolism and male-biased social deficits (41). Human observational studies connect maternal body mass index, gestational weight gain, infant gut microbiota, milk microbiota, SCFAs, and developmental outcomes, but residual confounding and reverse causation remain important limitations (42–44).

## 4 Dietary modulation of the maternal gut microbiome and cardiometabolic pathways

Maternal diet is a major (45) modifiable exposure that can shape microbial substrates and metabolic outputs during pregnancy. Diets high in saturated fat, refined carbohydrate, and ultra-processed foods have been associated in experimental and observational studies with lower microbial diversity, altered barrier function, and pro-inflammatory metabolic profiles, whereas fiber-rich and plant-diverse patterns support saccharolytic fermentation and SCFA production (46–48). Importantly, maternal diet and maternal adiposity are correlated; studies must separate their independent and interacting effects.

Evidence from animal models suggests that fermentable fiber can attenuate some maternal-diet-associated effects on offspring neurodevelopment and metabolism. High-fiber or inulin-containing diets have increased SCFA production and beneficial taxa and improved selected behavioral or inflammatory outcomes in offspring, while butyrate supplementation has reversed some diet-induced neurocognitive deficits (49–51). Translation to human pregnancy remains uncertain because doses, microbial ecosystems, and developmental timing differ substantially.

Overall, the most defensible clinical message is to prioritize established healthy dietary patterns rather than individual microbial taxa. Diets rich in vegetables, fruit, whole grains, legumes, nuts, and other sources of fiber and polyphenols may support microbial functional diversity and cardiometabolic health, but evidence is insufficient to prescribe a specific “microbiome diet” for pregnant women with obesity. Future trials should measure dietary adherence, microbial function, metabolites, and maternal and offspring cardiometabolic outcomes in the same protocol. Selected human evidence is summarized in Table 1.

**Table 1:** Selected human evidence linking maternal metabolic status, the maternal or infant microbiome, and related pathways.

Study	Design and population	Microbiome-related observation	Clinical interpretation
Koren et al. (12)	Longitudinal human pregnancy cohort	Gestational shifts in community structure and metabolic potential were reported.	Influential evidence for pregnancy-associated remodeling, but not a universal pregnancy signature.
Gomez-Arango et al. (13)	Human pregnancy cohort	Maternal microbial features were associated with metabolic measures and adiposity.	Observational association; directionality and independence from diet require caution.
Stanislawski et al. (14)	Human mother-infant cohort	Maternal obesity was associated with context-dependent differences in infant gut microbiota.	Delivery mode, antibiotics, feeding and shared environment limit causal attribution.
Lotankar et al. (15)	Metagenomic study in pregnant women with overweight or obesity	Microbial functional potential, including butyrate-related pathways, was associated with insulin resistance and lipid measures.	Supports function-focused hypotheses; does not establish mediation.
Hu et al. (16)	Human maternal-placental study	Maternal intestinal microbial features correlated with placental oxidative stress and redox measures.	Biologically plausible gut-placenta link; causal direction remains unresolved.

## 5 Nutritional and microbiome-targeted interventions

Preclinical studies suggest that prebiotics and selected probiotics can modify obesity-associated microbial and metabolic phenotypes during pregnancy. For example, oligofructose supplementation in obese pregnant rodents increased *Bifidobacterium* abundance and improved selected metabolic outcomes in mothers and offspring (47). However, animal findings should be viewed as hypothesis-generating rather than direct evidence for routine supplementation in human pregnancy.

Human trial results are mixed. In a large randomized, placebo-controlled trial of women with overweight or obesity, fish oil and/or probiotic supplementation was safe and well tolerated but did not reduce gestational diabetes or improve glucose metabolism (52). This contrasts with some earlier, smaller studies of probiotic-supplemented dietary counseling, illustrating that benefits may depend on strain, baseline risk, diet, timing, and outcome definition.

Long-term follow-up of a perinatal *Lactobacillus rhamnosus* GG intervention suggested moderation of excessive weight gain during early childhood, particularly among children who later became overweight, but it did not establish durable prevention of obesity through 10 years (53). Such findings are promising but require replication in trials designed specifically for maternal obesity and offspring cardiometabolic outcomes.

Next-generation probiotics are also being investigated. Preclinical studies outside pregnancy show that pasteurized *Akkermansia muciniphila* or its membrane components can improve metabolic phenotypes in obese and diabetic mice (54), and proof-of-concept trials in non-pregnant adults suggest acceptable safety and potential metabolic effects (55). These findings cannot yet be extrapolated to pregnancy, where maternal and fetal safety require dedicated evaluation.

Prebiotic fibers such as inulin can increase bifidobacteria and SCFA production, providing plausible mechanisms for effects on barrier integrity, inflammation, and glucose homeostasis (56). Nevertheless, pregnancy-specific evidence remains limited, and gastrointestinal tolerance, dose, background diet, and interindividual microbiome variation must be considered.

In summary, probiotics, prebiotics, and synbiotics are promising research tools but are not interchangeable interventions. Current evidence does not support broad claims that they prevent gestational diabetes or intergenerational obesity. Future trials should prespecify strains and doses, report safety, characterize baseline diet and microbiota, and include clinically meaningful maternal and offspring outcomes. Selected intervention evidence is summarized in Table 2.

**Table 2:** Selected dietary, lifestyle, probiotic, and prebiotic intervention evidence relevant to maternal obesity and pregnancy.

Study/evidence	Intervention and population	Main findings	Interpretation
GLOW/LIFE-Moms (106, 107)	Structured diet and lifestyle support during pregnancy in women with overweight or obesity	The interventions reduced or attenuated excess gestational weight gain.	Supports implementation of evidence-based lifestyle care; a microbiome mechanism was not tested.
DALI Lifestyle Study (108)	Diet and/or physical-activity counseling in pregnant women with overweight or obesity	Counseling influenced gestational weight gain and metabolic risk.	Supports lifestyle-focused prenatal care; a microbiome mechanism was not tested.
Whole-grain pregnancy trial (104)	Whole-grain versus refined-grain diet during pregnancy	Maternal anal and vaginal microbial communities differed between diets.	Shows diet-responsive microbial variation; clinical mediation was not established.
Fish oil/probiotic trial (52)	Probiotics, with or without fish oil, in pregnant women with overweight or obesity	The intervention did not significantly reduce gestational diabetes or improve glucose metabolism and was reported as well tolerated.	Does not support routine probiotic use for gestational diabetes prevention.
Prenatal multistrain probiotic trial (120)	Prenatal multistrain probiotic in women with obesity	The intervention influenced infant gut microbiome development but did not establish prevention of maternal or offspring metabolic disease.	Microbial effects do not by themselves demonstrate clinical benefit.
Perinatal L. rhamnosus GG follow-up (53)	Perinatal L. rhamnosus GG with childhood follow-up	Early excessive weight gain may have been moderated, but durable prevention of childhood obesity was not established.	Hypothesis-generating and not specific to maternal obesity.
Oligofructose rodent model (47)	Oligofructose in an obese pregnant rodent model	Bifidobacterium abundance and selected maternal and offspring metabolic outcomes changed.	Preclinical evidence cannot be extrapolated to routine pregnancy care.

## 6 Maternal microbial exposure and early-life gut assembly

Maternal microbial strains and exposures contribute to early infant gut assembly through several routes, including contact with maternal fecal, skin, oral, and milk-associated communities and the shared environment. Here, vertical transmission refers to maternal-to-infant acquisition of strains or microbial material, not the transfer of an intact maternal community. Strain-level studies indicate that maternal gut strains, particularly Bacteroides and Bifidobacterium, are important early colonizers, whereas cesarean delivery is associated with delayed acquisition of some maternal gut microbes and greater exposure to skin- and hospital-associated organisms (57–59).

Infants born to mothers with gestational diabetes or excessive gestational weight gain have shown differences in microbial diversity, taxa, and functional pathways, some of which are associated with later metabolic phenotypes (27, 60–62). However, maternal glycemia, body mass index, diet, antibiotics, delivery mode, and infant feeding are tightly correlated, limiting causal attribution.

Maternal metabolic and inflammatory status may affect microbial exposure and neonatal gut maturation indirectly through placental function, gestational age, delivery practices, and milk composition. Evidence does not currently show that maternal obesity consistently prevents transfer of specific organisms such as *Bifidobacterium longum* or *Akkermansia muciniphila*. Claims about taxon-specific transmission therefore require strain-resolved, longitudinal data.

Whether a resident placental or fetal microbiome exists in healthy pregnancy remains controversial. Low-biomass samples are highly vulnerable to contamination, and current evidence suggests that viable bacterial colonization *in utero* is absent or highly limited rather than a routine route of gut colonization (63). Prenatal microbiome-related influences, if present, may instead operate through microbial metabolites, immune signals, or occasional microbial exposure. Accordingly, infant gut colonization should be described as beginning predominantly during and after birth.

### 6.1 Mode of delivery and early colonization

#### 6.1.1 Vaginal vs. cesarean delivery

Delivery mode is consistently associated with early infant gut microbiome development, but the mechanism is more complex than acquisition of a vaginal community. Vaginal birth increases exposure to maternal fecal and perineal microbes, whereas cesarean delivery reduces contact with maternal gut strains and increases exposure to skin, hospital, antibiotic, and perioperative factors (57, 58).

Longitudinal studies show that cesarean-born infants often have delayed *Bacteroides* colonization, lower early microbial diversity, and greater abundance of opportunistic hospital-associated organisms; some differences diminish during infancy, whereas others persist longer (58, 64, 65). Breastfeeding, antibiotic exposure, household environment, and complementary feeding modify these trajectories.

Strain-level analyses support reduced maternal gut strain transfer after cesarean delivery (59). Recent cohorts also indicate that infant sex, parity, geography, and socioeconomic context interact with delivery mode (66–68). These findings argue against treating delivery mode as an isolated or deterministic exposure.

#### 6.1.2 Controversial interventions: vaginal seeding

Vaginal microbiota transfer (“vaginal seeding”) has been proposed to partially restore microbial exposures after cesarean delivery. Pilot and randomized studies report increased engraftment of selected maternal strains and shifts in infant microbial profiles, but restoration is incomplete and long-term health benefits have not been established (69, 70).

Because vaginal seeding may transmit pathogens and has uncertain clinical benefit, it should remain an experimental procedure conducted under rigorous screening and research oversight. Current evidence instead supports established practices such as appropriate antibiotic stewardship, breastfeeding support, and avoidance of non-medically indicated cesarean delivery when clinically feasible.

### 6.2 Breastfeeding and milk-associated microbiota

After birth, breastfeeding influences infant gut development through milk-associated microbes, human milk oligosaccharides (HMOs), immune factors, metabolites, and repeated contact with maternal skin and the household environment. These lactational influences are related to, but distinct from, delivery-associated acquisition of maternal gut strains.

Milk-associated Lactobacillus, Bifidobacterium, Staphylococcus, and Streptococcus have been described in human milk and may contribute to infant community assembly, although the relative contribution of viable milk organisms remains uncertain (71). Because milk is a low-biomass sample, contamination control, collection method, lactation stage, antibiotic exposure, and feeding practices are important considerations.

Maternal obesity has been associated with differences in milk microbial composition, but findings are heterogeneous and should not be interpreted as a consistent obesity-specific milk microbiota. Reported associations are sensitive to sampling (72–75), low-biomass contamination, delivery mode, antibiotics, lactation stage, geography, and other maternal characteristics (29, 76–78).

Longitudinal studies suggest that milk microbial communities change across lactation and may be associated with infant gut development (79, 80). The proposed entero-mammary route is biologically plausible but incompletely demonstrated in humans (81). These observations do not show that maternal obesity directly changes infant outcomes through milk microbes or that probiotic supplementation normalizes the milk microbiota.

### 6.3 Human milk oligosaccharides, milk metabolites, and infant gut development

#### 6.3.1 HMOs and infant microbial development

HMOs are indigestible milk glycans that serve as selective substrates for infant-associated bacteria, particularly bifidobacteria, and can influence microbial metabolism and immune development. Their effects depend on the specific HMO and the metabolic capacity of the colonizing strains. Evidence linking HMO (82–86) profiles with infant microbial community structure is substantial, but longer-term cardiometabolic implications remain uncertain.

#### 6.3.2 Maternal metabolic status and HMO variation

HMO concentrations vary considerably among individuals. Maternal adiposity and glycemic status (87–89) have been associated with selected HMOs and the broader milk metabolome, but findings are not uniform. Secretor status, genetics, lactation stage, diet, and analytical method are major determinants and potential confounders. Maternal obesity (72–75) should therefore be considered one possible correlate of HMO variation rather than a uniform cause of an adverse HMO profile.

#### 6.3.3 Dietary and metabolic determinants of HMOs

HMOs are also detectable in maternal circulation during pregnancy, and their concentrations have been associated with glucose responses and insulin sensitivity (90). Gestational diabetes has been associated with differences in HMO concentrations across lactation, although findings are inconsistent (91, 92). Observational studies link maternal dietary factors (93, 94) with selected HMO abundances, but intervention studies are needed to determine whether diet-associated changes in HMOs meaningfully alter infant microbial function or health (95).

## 7 Longitudinal outcomes in offspring

### 7.1 Metabolic outcomes: childhood obesity and insulin resistance

Early-life gut microbiome development is associated with later adiposity and metabolic health, particularly through pathways involving energy metabolism, gut barrier function, and immune maturation (96, 97). However, direct evidence linking maternal-obesity-associated microbiome differences to childhood insulin resistance remains limited. Longitudinal studies with repeated maternal and child sampling, strain-level analysis, and objective cardiometabolic outcomes are needed to distinguish mediation from shared genetic, dietary, and environmental influences.

### 7.2 Immune dysregulation and inflammatory phenotypes

Infant microbial colonization contributes to immune-system maturation, but the reviewed evidence does not establish that maternal-obesity-associated microbial differences independently drive a specific inflammatory phenotype. Studies in preterm or infection-exposed populations and mechanistic animal models help identify plausible immune–microbiome pathways, yet their generalizability to term infants born to mothers with obesity is uncertain (98–100). Sex-specific immune and endocrine responses may further modify associations (101).

### 7.3 Neurodevelopmental outcomes and sex-specific vulnerabilities

Neurodevelopment is sensitive to prenatal immune, metabolic, and dietary exposures, and infant microbial and metabolite profiles have been associated with later cognitive and behavioral outcomes (43, 102). Nevertheless, the current literature does not demonstrate that the maternal-obesity microbiome pathway independently causes neurodevelopmental disorders. Future analyses should use longitudinal causal frameworks while avoiding overinterpretation of predictive associations (103).

## 8 Clinical and public health implications

### 8.1 Evidence-based prenatal care and microbiome research

Current clinical care should remain grounded in established prenatal nutrition and lifestyle guidance. Individualized dietary counseling, support for appropriate gestational weight gain, breastfeeding support, prudent antibiotic use, and avoidance of non-medically indicated cesarean delivery where feasible are justified by broader maternal and infant health evidence. A fiber-rich, plant-diverse dietary pattern may also support favorable microbial functions, but microbiome changes should be regarded as exploratory outcomes rather than the basis for prescribing a specific pregnancy microbiome diet.

Pregnancy trials show that dietary quality can alter maternal microbial communities. For example, a comparison of whole-grain and refined-grain diets identified differences in maternal anal and vaginal microbial profiles (104), and secondary analyses of the IMPACT-BCN trial associated Mediterranean-diet and mindfulness interventions with maternal gut and vaginal microbiota (105). These findings do not establish that microbial changes mediated clinical benefits.

By contrast, routine microbiome testing and the use of probiotics, prebiotics, or synbiotics specifically to prevent gestational diabetes or offspring obesity are not supported by consistent pregnancy-specific evidence. Next-generation probiotics, microbiome-guided precision nutrition, vaginal seeding, and fecal microbiota transplantation for metabolic prevention should remain within appropriately governed research settings. These strategies differ substantially in rationale, evidence, and safety considerations and should not be presented as interchangeable extensions of dietary counseling.

### 8.2 Integration into guidelines for managing maternal obesity

Scaling evidence-based lifestyle interventions into public health policy is essential for addressing maternal obesity. The GLOW randomized trial demonstrated that a telehealth-delivered lifestyle program within a large health system reduced excess gestational weight gain among women with overweight and obesity (106). These findings illustrate how structured interventions can be feasibly integrated into routine prenatal care at the systems level.

Similarly, results from the LIFE-Moms consortium, which tested multiple lifestyle intervention models across diverse U.S. sites, confirmed that structured counseling effectively attenuated gestational weight gain in women with BMI ≥ 25 (107). The multi-site design of this trial highlights the adaptability of such interventions to a wide range of healthcare settings, strengthening the evidence base for policy-level adoption.

The DALI Lifestyle Study extended this framework internationally. Conducted across nine European countries, this multicenter randomized trial demonstrated that diet and/or physical activity counseling in women with overweight or obesity influenced gestational weight gain and metabolic risk, underscoring the cross-cultural applicability of lifestyle-focused guidelines (108).

Longer-term maternal and offspring outcomes provide further rationale for integration. Dodd et al. found that antenatal dietary interventions in women with overweight and obesity had measurable effects on infant BMI and growth trajectories (109). Such findings emphasize the intergenerational benefits of embedding lifestyle interventions into maternal health guidelines.

Finally, the UPBEAT trial follow-up showed that women receiving a lifestyle intervention during pregnancy reported improved diet quality sustained up to three years postpartum, with associated effects on offspring cardiometabolic outcomes (110). This demonstrates that pregnancy is a critical window for intervention with lasting impact, reinforcing calls for guideline updates that prioritize dietitian-led counseling and lifestyle modification as standard components of prenatal care.

## 9 Investigational and emerging strategies

### 9.1 Next-generation probiotics and precision nutrition

Next-generation probiotics and precision nutrition are promising but remain investigational in pregnancy. A proof-of-concept trial in non-pregnant adults with overweight and insulin resistance found that pasteurized *Akkermansia muciniphila* was well tolerated and associated with improvements in selected metabolic measures (55). This study supports biological plausibility, not pregnancy-specific efficacy or safety.

Subsequent adult trials have continued to evaluate *A. muciniphila* for glycemic and inflammatory outcomes (111). Pregnancy, however, involves distinct immune and metabolic physiology; interventions should not be recommended until reproductive toxicity, maternal safety, fetal outcomes, and strain- or product-specific effects are established.

Precision-nutrition studies in non-pregnant adults show that personalized dietary programs incorporating microbiome and postprandial data can improve selected cardiometabolic outcomes (112). Such approaches may eventually inform maternal care, but algorithms require validation in diverse pregnant populations and must demonstrate benefit beyond standard dietitian-led counseling.

The PREDICT-1 study demonstrated substantial interindividual variability in postprandial glycemic and lipid responses and showed that host and microbiome features can contribute to prediction (113). Prediction does not necessarily identify causal microbial targets, and pregnancy-specific models will require calibration for gestational age, adiposity, insulin resistance, medications, and dietary intake.

Microbiome-guided diets have also been tested in conditions such as irritable bowel syndrome (114). These studies demonstrate technical feasibility but are not evidence for efficacy in maternal obesity. Clinical implementation should therefore await pregnancy-specific randomized trials, transparent algorithms, and safety monitoring.

Together, next-generation probiotics and precision nutrition illustrate two complementary research directions: targeted manipulation of specific microbial functions and individualized dietary support based on host–microbiome responses. Both require substantially stronger pregnancy-specific evidence before routine use.

### 9.2 Potential role of fecal microbiota transplantation (FMT)

Fecal microbiota transplantation (FMT) is an established treatment for selected cases of recurrent *Clostridioides difficile* infection, but its use for maternal obesity or routine infant microbiome restoration is experimental. In a proof-of-concept study, carefully screened maternal fecal microbiota transfer to cesarean-born infants accelerated development of a microbiota resembling that of vaginally born infants (115). The study was small and was not designed to establish long-term clinical benefit.

Reports of FMT during pregnancy are limited to exceptional clinical situations, such as recurrent *C. difficile* infection or severe inflammatory bowel disease when standard options are inadequate (116). Case reports can inform feasibility but cannot establish safety for broader indications.

Washed microbiota transplantation has likewise been described in a pregnant patient with hypervirulent *C. difficile* infection (117). These reports should not be generalized to microbiome treatment of obesity because donor screening, pathogen transmission, antimicrobial resistance, and maternal–fetal safety remain major concerns.

Evidence from non-pregnant populations confirms that FMT can be effective for recurrent *C. difficile* infection (118), but efficacy for metabolic disorders is inconsistent and product standardization remains challenging.

Donor-derived strains may persist and, in rare circumstances, be detected across generations after maternal FMT (119). This possibility underscores the need for long-term surveillance and careful ethical review rather than supporting routine intergenerational microbiome manipulation.

Accordingly, FMT should not currently be proposed as a preventive or metabolic intervention for maternal obesity or healthy neonates outside approved research protocols. Pregnancy-specific registries and controlled studies are needed to define indications, safety, and long-term outcomes.

## 10 Discussion

Maternal obesity is associated with metabolic inflammation and with context-dependent differences in the maternal gut microbiome during pregnancy. Across the reviewed literature, the most reproducible signal is not a single taxonomic profile but variation in microbial functions and host-microbe outputs, including butyrate-producing potential, SCFAs, bile-acid metabolism, and endotoxin-related signaling. Even for these pathways, human evidence is largely associative. Pregnancy-specific trials and prospective cohorts therefore carry greater interpretive weight than animal experiments or studies in non-pregnant adults. The proposed pathways are summarized in Figure 1.

**Figure 1:**
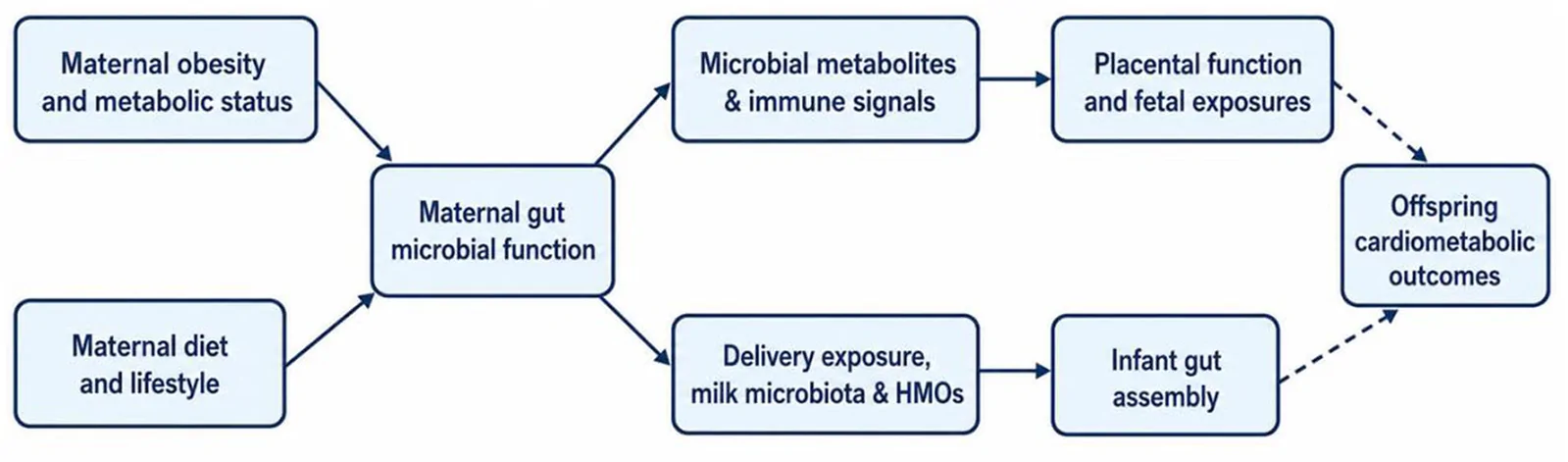
Proposed pathways linking maternal obesity and diet with microbial functions, placental and lactational exposures, infant gut assembly, and offspring cardiometabolic outcomes. Solid arrows indicate comparatively better-supported associations; dashed arrows indicate proposed pathways that require confirmation in longitudinal, experimental, or mediation-based studies. —, comparatively better-supported association. —-, proposed pathway requiring causal or mediation evidence. Important sources of confounding include genetics, medications and antibiotics, geography, socioeconomic conditions, delivery care, feeding and the shared post-natal environment.

Maternal microbial and metabolic exposures may contribute to placental function and early infant gut assembly through metabolites, immune signaling, delivery-related exposure, breastfeeding, HMOs, antibiotics, and the shared environment. Evidence is strongest for associations between maternal metabolic status, microbial features, and early infant colonization. Direct mediation of later childhood obesity, insulin resistance, immune outcomes, or neurodevelopment by the maternal microbiome remains unconfirmed and requires longitudinal studies designed for causal and mediation analysis.

The clinical implications follow this evidence hierarchy. High-quality, individualized dietary and lifestyle care is appropriate because its benefits do not depend on proving a microbiome mechanism. In contrast, microbiome-targeted supplements, next-generation probiotics, precision-nutrition algorithms, vaginal seeding, and FMT lack sufficient pregnancy-specific evidence for routine metabolic prevention. The most tractable next step is to test clearly specified dietary or microbial interventions while measuring adherence, microbial function, metabolites, safety, and meaningful maternal and offspring outcomes in the same study.

## 11 Study strengths and limitations

This narrative review integrates human cohorts, clinical trials, animal models, and mechanistic studies across maternal obesity, diet, microbial metabolites, placental pathways, early-life colonization, and offspring health. Its broad scope permits synthesis across disciplines and highlights where dietary and microbiome-related mechanisms may intersect with cardiometabolic programming.

Several limitations should temper interpretation. The search was structured but not conducted as a systematic review, and study selection and synthesis were not preregistered. The evidence is heterogeneous in population characteristics, timing of sampling, dietary assessment, sequencing platform, bioinformatic methods, and control of confounding. Many studies are small or cross-sectional, and animal or non-pregnant adult findings are not directly generalizable to pregnancy.

In addition, taxonomic associations are often inconsistent, low-biomass samples are vulnerable to contamination, and several intervention findings are strain- and context-specific. Consequently, this review emphasizes associations and biological plausibility rather than confirmed causal pathways or clinical efficacy.

## 12 Conclusion

Maternal obesity, diet, and the gut microbiome form an interconnected but incompletely resolved system that may contribute to maternal metabolic health and offspring cardiometabolic programming. The strongest current clinical strategy is high-quality, individualized dietary and lifestyle care. Microbiome measures and targeted microbial interventions are promising research tools, but they require pregnancy-specific validation, rigorous safety assessment, and demonstration of clinically meaningful benefit before routine implementation. Clarifying these pathways may ultimately help reduce intergenerational cardiometabolic risk.

## 13 Future directions

Future studies should begin before conception or in early pregnancy and include repeated maternal and infant sampling, detailed dietary assessment, and strain-level or function-resolved microbiome methods integrated with metabolomics and placental phenotyping. Longitudinal mediation analyses are needed to test whether microbial functions or metabolites explain associations between maternal metabolic status and offspring outcomes. Pregnancy-specific randomized trials should prespecify strain or intervention composition, dose, adherence, safety, and clinically meaningful maternal and offspring endpoints. Progress will also depend on diverse populations, standardized sampling and sequencing methods, transparent analytical plans, replication, and long-term offspring follow-up.
